# A Comparative Study of the Biological Properties of *Eugenia uniflora* L. Fruits and Leaves Related to the Prevention of Cardiovascular Diseases

**DOI:** 10.3390/life15020147

**Published:** 2025-01-22

**Authors:** Jéssica Gonçalves, Nance Hontman, Rosa Perestrelo, José S. Câmara

**Affiliations:** 1 CQM—Centro de Química da Madeira, Universidade da Madeira, Campus da Penteada, 9020-105 Funchal, Portugal; jessica.goncalves@staff.uma.pt (J.G.); nance.hontman@staff.uma.pt (N.H.); rmp@staff.uma.pt (R.P.); 2 Departamento de Química, Faculdade de Ciências Exatas e Engenharia, Universidade da Madeira, Campus da Penteada, 9020-105 Funchal, Portugal

**Keywords:** *Eugenia uniflora* L., polyphenols, biological activities, cardiovascular diseases

## Abstract

Cardiovascular diseases (CVDs) remain the leading cause of death globally, emphasizing the need for effective preventive strategies. Plant-based foods, rich in phytochemicals, offer a promising potential in CVD prevention. This study investigated the antioxidant, anti-inflammatory, and antihypertensive properties of two *Eugenia uniflora* L. varieties (orange and purple pitanga) and their leaves. Their antioxidant activity was assessed using 2,2-diphenyl-1-picrylhydrazyl (DPPH) free radical scavenging and 2,2′-azino-bis(3-ethylbenzothiazoline-6-sulfonic acid) (ABTS) radical cation scavenging activity assays, while their antihypertensive activity was evaluated through angiotensin-converting enzyme (ACE) inhibition. Their anti-inflammatory potential was determined via protein denaturation inhibition. Both fruit varieties exhibited similar bioactivities, with the purple variety showing a slightly higher activity, except in the DPPH and ABTS assays. The leaves consistently demonstrated the lowest activities across all assays. Free polyphenols, dominated by gallic acid, were quantified using µ-QuEChERS followed by ultrahigh-performance liquid chromatography (UHPLC-PDA). The orange variety contained the highest concentration of gallic acid (13.1 mg/100 g DW). These findings highlight the potential of *Eugenia uniflora* L. extracts as natural antioxidant, anti-inflammatory, and antihypertensive agents, suggesting their value in food, pharmaceutical, and cosmetic applications for promoting human health and preventing CVDs.

## 1. Introduction

*Eugenia uniflora* L. is an indigenous Brazilian plant of the *Myrtaceae* family. The *Myrtaceae* family is one of the major commercial fruit families in the world [1]. Representative plants of the *Myrtaceae* family have significant agro-industrial potential in addition to ecological significance [2]. They are used in folk medicine to prevent and treat some symptoms related to hypertension, flu, fever, cough, and diarrhea [3]. The medicinal properties of *Eugenia uniflora* L. are widely established, since it has anti-inflammatory [4], antihypertensive, and antioxidant properties [5], which prevent lipid peroxidation and the formation of free radicals. Additionally, it possesses antiproliferative, antiviral, antifungal, and antibacterial properties [5]. These properties have a great impact on the prevention of cardiovascular diseases (CVDs), cancer, and neurodegenerative diseases [5]. Recent research suggests that *Eugenia uniflora* L. is a viable natural source of phytochemicals used in the formulation of novel drugs that target the central nervous system (CNS) [6,7]. Some studies have reported the antihypertensive activity of *Eugenia uniflora* L. [8,9], resulting in a reduction in ROS overproduction, the normalization of serum lipids (cholesterol, LDL, and HDL), the regulation of anti-inflammatory and anti-apoptosis processes that protect against myocardial injuries, the inhibition of ACE activity (overactivation of the RAAS causes cardiovascular dysfunction), and consequently a lower blood pressure [10,11]. The pharmacological basis of this practical usage has been investigated in several studies. Anconatani et al.’s [8] work reported that *Eugenia uniflora* L. has a hypotensive action that is mediated by direct vasodilatory activity. A diuretic effect was also reported by the same authors, which may be brought on by an increase in renal blood flow [8].

Diet is a fundamental factor in disease prevention and overall health [12]. The intricate relationship between nutrition and health means that a well-balanced diet can be instrumental in warding off illness, while dietary habits profoundly impact the risk factors associated with various diseases [13]. According to recent studies, the Mediterranean diet, based on fruit and vegetable consumption, is the most cardioprotective, because it contains a lot of bioactive compounds like fiber, phytosterols, polyunsaturated fatty acids, polyphenols, and vitamins that have anti-inflammatory, antithrombotic, and antioxidant properties that help to postpone the onset and progression of CVDs [14]. In this way, it is possible to identify potential targets (dietary patterns, single foods, or individual nutrients) to prevent CVDs and quantify the magnitude of these beneficial effects [15]. Some studies have demonstrated the preventive benefits of a polyphenol-rich diet against most chronic illnesses [16]. This has also been associated with a reduced CVD incidence [17] by improving the function of the inner lining of the heart and blood vessels, increasing HDL cholesterol, and decreasing LDL cholesterol, thus promoting antiplatelet and anti-inflammatory effects [18]. However, the type, amounts, and bioavailability of polyphenols, which are influenced by their source, food matrix, processing, digestion, and cellular metabolism, are closely linked to the magnitude of their health effects [19]. Plant polyphenols are a suitable alternative to synthetic preservative agents, with antioxidant and antimicrobial properties [20]. Several drawbacks, including a low solubility and stability during food processing and storage, a lack of standardization, and undesirable organoleptic properties, limit their applications in the food industry [21].

**Figure 1:**
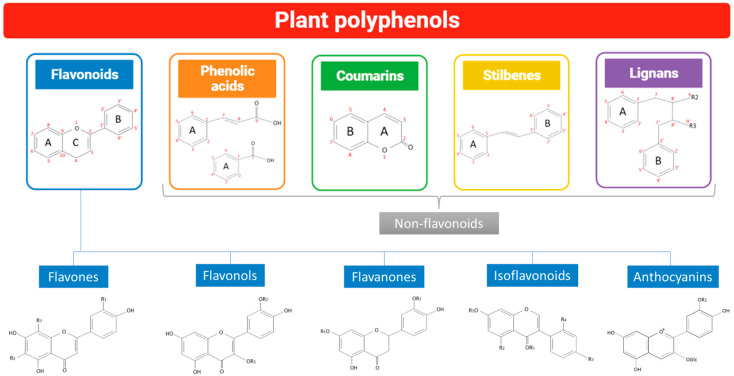
Polyphenol classification into flavonoids (flavones, flavonols, flavanones, isoflavonoids, and anthocyanins) and non-flavonoids (phenolic acids, coumarins, stilbenes, and lignans).

Dietary polyphenols also aid in reducing systemic inflammation, insulin resistance, blood pressure, and lipid profiles. *Trans*-resveratrol, a stilbene, and the flavonoid quercetin have both been related to a better cardiovascular health, as shown in Figure 1 [22]. Polyphenol intake is important for maintaining a good health. However, the metabolism, transportation, and distribution to target organs are complex processes not yet completely understood. The bioaccessibility and bioavailability of polyphenols in the gastrointestinal system are the key determinants of their absorption. In contrast to bioavailability, which refers to a substance’s activity for digestion and dispersion by the body, bioaccessibility refers to the amounts of these compounds that are accessible for metabolic processes and can be modified by the interaction of polyphenols with dietary components [23]. Polyphenols often have a poor bioavailability since their metabolism is influenced by a variety of parameters, including their solubility, chemical structure, degree of polymerization, and interactions with other molecules, among others. Phases I and II of polyphenol metabolization, which occur in the cells of the liver and gut, respectively, may be split into two categories. Phase I of polyphenol oxidation, reduction, and hydrolysis results in modifications to the amino, carboxyl, and hydroxyl groups that make up their structure. On the other hand, in phase II, the chemicals’ toxicity is decreased, and they are eliminated by enzymatic processes [23].

Extraction techniques such as solid–liquid extraction [24], pressurized liquid extraction (PLE) [25], and ultrasound-assisted extraction (UAE) coupled with the HPLC-UV method [26] have been reported for the extraction of polyphenols from different samples. In the investigation carried out by Lazzarotto-Figueiró et al. [27], UAE was used to extract polyphenols from plants of the *Myrtaceae* family, including *Eugenia* species. Another study by Bagatini et al. [25] investigated the extraction of polyphenols from *Eugenia uniflora* L. leaves using aqueous infusion and PLE [25]. An emerging technique for extracting polyphenols from *Eugenia uniflora* L. leaves is energized dispersive guided extraction (EDGE^®^), which was developed by utilizing response surface methodology [28]. Some of these studies also used spectrophotometric tests to determine the polyphenolic content and antioxidant potential, such as Folin–Ciocalteu, 2,2-diphenyl-1-picrylhydrazyl (DPPH), and 2,2′-azinobis (3-ethylbenzothiazoline-6-sulfonic acid) (ABTS) [25,27,29]. Silva et al. [30] employed the QuEChERS extraction technique, combined with dispersive solid-phase extraction (dSPE), to extract polyphenols from tropical fruits, including orange pitanga. The HPLC-DAD-ESI-MS^n^ technique allowed them to identify myricetin arabinopyranoside and quercetin rhamnose as the most dominant compounds.

The principal aim of this work is to deepen our knowledge of the contributions of food to CVD prevention, with a particular focus on *Eugenia uniflora* L. fruits, often referred to as “pitanga”. The study will particularly compare the biological properties, namely the antioxidant, anti-inflammatory, and antihypertensive activity, of different varieties of the pitanga fruit (orange and purple) and leaves of *Eugenia uniflora* L. to evaluate their potential in CVD prevention. In addition, we aim to identify and quantify the polyphenols from the investigated matrices using the µ-QuEChERS/UHPLC-PDA method.

## 2. Materials and Methods

### 2.1. Reagents and Standards

All used reagents and standards were of analytical grade. Potassium persulfate (K_2_S_2_O_8_, 99.0%) and potassium phosphate dibasic trihydrate (K_2_HPO_4_•3H_2_O) were acquired from Merck^®^ (Buchs, Switzerland). Sodium chloride (NaCl, 99.5%), trisodium citrate dihydrate (C_6_H_9_Na_3_O_9_, 99.0%), potassium dihydrogen phosphate (KH_2_PO_4_, 99.5%), N-[3-(2-furyl)acryloyl]-Phe-Gly-Gly (FAPGG), and formic acid (FA, CH_2_O_2_, 98.0%) were attained from Panreac Applichem (Barcelona, Spain). Aluminum chloride (AlCl_3_), potassium chloride (KCl, 99.5%), ethyl acetate (EtAc, C_4_H_8_O_2_, 99.7%), and quercetin (C_15_H_10_O_7_•2H_2_O, 99.1%) were supplied by Riedel-de Haën^®^ (Seelze, Germany). 2,2′-Azino-bis-(3-ethylbenzothiazoline-6-sulfonic acid) diammonium salt (ABTS, C_18_H_24_N_6_O_6_S_4_, 98.0%), resveratrol (C_14_H_12_O_3_, 99.0%), and kaempferol (C_15_H_10_O_6_, 97.0%) were acquired from Sigma-Aldrich (Buchs, Switzerland). Sodium carbonate (Na_2_CO_3_, 99.7%) was supplied by Labsolve^®^ (Lisbon, Portugal). Syringaldehyde was acquired from Acros Organics (Geel, Belgium). HPLC-grade ACN (CH_3_CN) and methanol (MeOH, CH_3_OH) were acquired from Fisher Scientific (Loughborough, UK), as were angiotensin-converting enzyme (ACE, from human, 95.0%), hydrochloric acid (HCl, 37.0%), and trisodium citrate dihydrate (C_6_H_5_Na_3_O_7_•2H_2_O, 99%). Folin–Ciocalteu solution, 2,2-diphenyl-1-picrylhydrazyl (DPPH, C_18_H_12_N_5_O_6_), 6-hydroxy-2,5,7,8-tetramethylchromane-2-carboxylic acid (Trolox, C_14_H_18_O_4_, 98.0%), gallic acid (C_7_H_6_O_5_, 98.0%), and cinnamic acid (C_9_H_8_O_2_, 99%) were supplied by Fluka (Munich, Germany). In addition, 2 mL DisQuE™ dSPE tubes containing the sorbents (150 mg of MgSO_4_ and 25 mg PSA), which were utilized in the μ-QuEChERS clean-up setup, were acquired from Waters (Milford, MA, USA). Ultrapure water (H_2_O) (18 MΩ cm) was attained from a Milli-Q water purification system (Millipore, Burlington, MA, USA).

### 2.2. Samples and Sample Treatment

**Figure 2:**
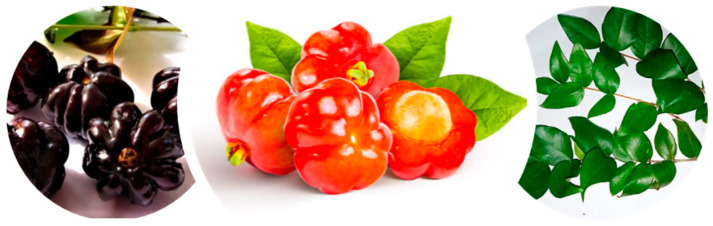
Image of the analyzed samples, fruits from orange and purple varieties of *Eugenia uniflora* L., as well as the leaves of the orange pitanga.

The *Eugenia uniflora* L. fruits used in this study, from both orange and purple varieties, were purchased in Mercado dos Lavradores (Madeira Island, Portugal) at a mature stage, as used for consumption, in July 2023. After washing, the pitanga seeds were removed from the fruit. Then, fruits from the orange and purple varieties of *Eugenia uniflora* L. and leaves from the orange pitanga were lyophilized, powdered, and stored in amber glass vials at room temperature (25 ± 1 °C) until extraction. Figure 2 depicts a picture from the different analyzed samples.

### 2.3. Extraction by µ-QuEChERS

**Figure 3:**
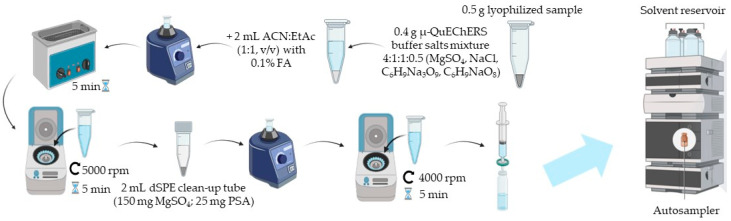
Schematic overview of the analytical methodology used in the study.

The μ-QuEChERS extraction technique used for the isolation of polyphenols from *Eugenia uniflora* L. leaves and fruits was according to the method described by Casado et al. [31]. Briefly, 0.5 g of lyophilized sample was added to 0.4 g of the μ-QuEChERS buffer salts mixture in a ratio of 4:1:1:0.5 (MgSO_4_, NaCl, C_6_H_9_Na_3_O_9_, C_6_H_9_NaO_8_, respectively). Then, 2 mL of an ACN:EtAc solution (1:1, *v*/*v*) containing 0.1% FA was added, and the flask was vortexed. The mixture was then subjected to ultrasonic agitation for 5 min and centrifuged for 5 min at 5000 rpm. The supernatant (~1000 μL) was then transferred to a 2 mL DisQueTM dSPE clean-up tube. Everything was vortexed before being centrifuged for 5 min at 4000 rpm. The extract was filtered using 0.22 μm PTFE syringe filters (BGB Analytik, Alexandria, VA, USA) into a vial and kept at −20 °C for analysis. The schematic procedure for the μ-QuEChERS extraction is presented in Figure 3.

### 2.4. Total Phenolic Content

**Figure 4:**
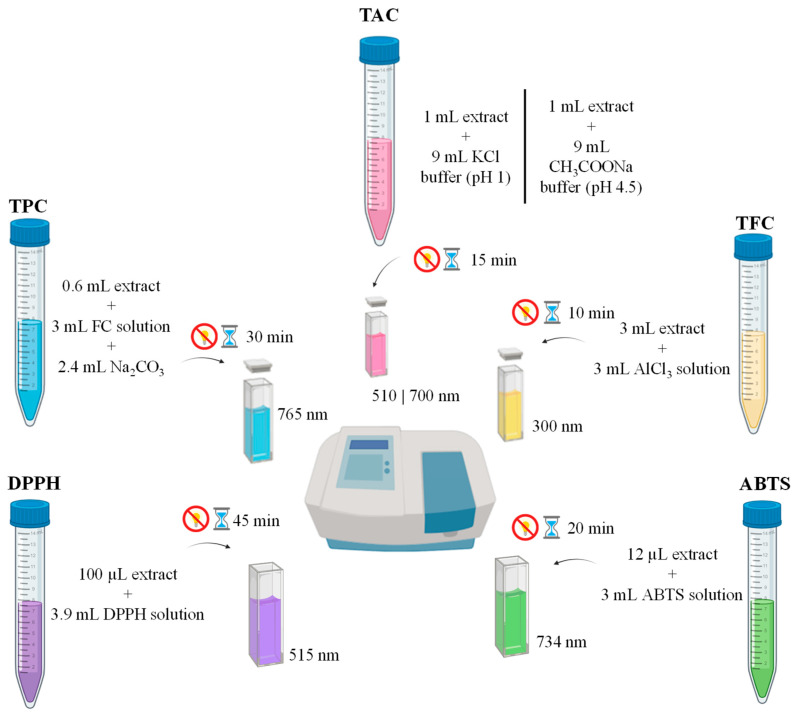
Schematic overview of the assays used for determination of total phenolic content (TPC), total flavonoid content (TFC), total anthocyanin content (TAC), and antioxidant activity (DPPH, ABTS assays).

The total phenolic content (TPC) of the *Eugenia uniflora* L. fruits and leaves extracts was determined using the Folin–Ciocalteu procedure described by Figueira et al. [32], with some modifications. Briefly, 3 mL of Folin–Ciocalteu solution (1:10 *v*/*v*) and 2.4 mL of 7.5% (*w*/*v*) Na_2_CO_3_ solution were added to 600 µL of extract, as shown in Figure 4. The mixture was homogenized and incubated for 30 min in the dark and at 25 ± 1 °C. After incubation, absorbance was measured using a UV–Vis spectrophotometer (Lambda 25, Perkin Elmer, Waltham, MA, USA) at a wavelength of 765 nm. The results are expressed in mg of gallic acid equivalents (GAE)/100 g of dried weight (DW) and were calculated using a calibration curve (R^2^ = 0.992) prepared with gallic acid standard (15 to 76 mg/L). TPC measurements were performed in triplicate.

### 2.5. Total Flavonoid Content

The total flavonoid content (TFC) was measured using the AlCl_3_ colorimetric assay described by Figueira et al. [32], with a few adjustments. In total, 3 mL of 2% *w*/*v* of AlCl_3_ in methanol was added to 3 mL of sample extract and incubated in the dark for 10 min at 25 ± 1 °C, and absorbance was measured at 300 nm, as shown in Figure 4. The results, expressed as mg of quercetin equivalents (QE)/100 g dry weight, were calculated using a calibration curve (R^2^ = 0.998) prepared with quercetin standard (5 to 40 mg/L). TFC measurements were performed in triplicate.

### 2.6. Total Anthocyanin Content

The total anthocyanin content (TAC) was determined by the pH differential method described by Sudarat et al. [33], with some modifications. For the assay, 500 µL of leaf and fruit extracts was added to a 5 mL volumetric flask to produce two dilutions of the extracts, one adjusted with a buffer pH of 1.0 (potassium chloride buffer) and the other to pH 4.5 (sodium acetate buffer). The solutions were left to equilibrate for 15 min. Absorbance was measured at 510 and 700 nm. Monomeric anthocyanin pigments were calculated using the following formula:

TAC (mg/L) = (A × MW × DF × 1000)/(ε × 1)

where MW = 449.2 g/mol, DF is the dilution factor, and ε = 26,900 L/mol·cm. The results are expressed as mg cyanidin-3-glucoside equivalents (C3GE)/100 g dry weight.

### 2.7. Determination of Biological Properties from Eugenia uniflora L. Fruits and Leaves

#### 2.7.1. 2,2-Diphenyl-1-pcrylhydrazyl Scavenging Assay

The 2,2-diphenyl-1-picrylhydrazyl (DPPH) test was carried out following Figueira et al. [32], with slight changes, to determine the free radical scavenging activity of the *Eugenia uniflora* L. fruits and leaf extracts under study. A total of 100 µL of the extracts was reacted with 3.9 mL of DPPH working solution (absorbance ~0.900 ± 0.01) for 45 min in the dark at 25 ± 1 °C, and absorbance was measured at 515 nm, as shown in Figure 4. The results, expressed as µM Trolox equivalents (TE)/100 g dry weight, were calculated using a calibration curve (25–600 µg/mL Trolox) and assessed in triplicate.

#### 2.7.2. 2,2′-Azino-bis(3-ethylbenzothiazoline-6-sulfonic) Acid Assay

The 2,2′-azino-bis(3-ethylbenzothiazoline-6-sulfonic) acid (ABTS) test was modified from the method published by Figueira et al. [32] to determine the antioxidant activity of the *Eugenia uniflora* L. leaf and fruit extracts against the stable ABTS^·+^ radical cation. ABTS^·+^ radical cations were generated by reacting ABTS (20 mM) with potassium persulfate (70 mM) and incubating in the dark for 16 h. The ABTS solution was diluted with PBS until an absorbance value of ~0.900 ± 0.01 was obtained. Then, 12 μL of the extracts was added to 3 mL of the diluted ABTS solution. The mixture was then homogenized and incubated for 20 min in the dark at 25 ± 1 °C, as shown in Figure 4. Absorbance was measured at 734 nm, and antioxidant activity is expressed as µM Trolox equivalents (TE)/100 g dry weight using a calibration curve (100–1500 µg/mL Trolox), with all measurements performed in triplicate.

#### 2.7.3. Antihypertensive Activity Assay

The antihypertensive activity was evaluated using the ACE inhibition activity assay reported by Figueira et al. [32], with some modifications. Briefly, 50 µL of FAPGG (2 mM) was diluted in 450 µL of Tris-HCl buffer (50 mM, with 300 mM NaCl and 0.1 M HCl at pH 8.3). After vortex homogenization (1 min), 400 µL of H_2_O was added, then 50 µL of extract, followed by homogenization, before adding 50 µL of ACE (0.5 U diluted from a 5 U stock solution in a potassium phosphate buffer—KH_2_PO_4_ 9.3 mM and K_2_HPO_4_ • 3H_2_O 0.7 M; with 300 mM NaCl at pH 8.3) and incubating for 3 min at 37 °C. Absorbance at 328 nm was recorded every 2 min for 20 min. The inhibition rate was calculated using the following formula:

% Inhibition ACE = (1 − (Activity with inhibition/Activity without inhibition)) × 100

to measure the enzymatic activity in the presence and absence of the inhibitor.

#### 2.7.4. Anti-Inflammatory Activity Assay

The anti-inflammatory activity was evaluated by analyzing the inhibitory activity of the extracts relative to protein denaturation, according to Gunathilake et al. [34], with some modifications. Briefly, 100 μL of extract was diluted in 4 mL of PBS (pH 6.4), mixed with 2% albumin, vortexed, and incubated at 37 °C for 30 min, followed by heating at 70 °C for 20 min. Absorbance was measured at 660 nm, and the percentage inhibition of protein denaturation was calculated as follows:

% Inhibition of protein denaturation = ((A_1_ − A_2_)⁄A_1_) × 100

where A_1_ is the control absorbance (PBS) and A_2_ is the sample absorbance. Aspirin was used as a standard.

### 2.8. UHPLC-PDA Conditions

A UHPLC system (Waters Ultra-High Performance Liquid Chromatography Acquity H-Class system) (Milford, MA, USA) equipped with a quaternary solvent manager (QSM), an Acquity sample manager (SM), a column heater, a degassing system, and a photodiode array detector (2996 PDA) was used for the chromatographic analysis of the *Eugenia uniflora* L. fruits and leaves. The column used to separate the analytes was an Acquity UPLC^®^ CSH™ C18 analytical column (2.1 mm × 150 mm, 1.7 μm particle size) (Waters, Milford, MA, USA). The analytes of interest were separated chromatographically at a column temperature of 40 °C, using a mobile phase of acidified water (0.1% FA) (solvent A) and ACN (solvent B), under the following conditions: 80% A (0 min), 60% A (3 min), 55% A (6 min), 30% A (7 min), 20% A (7.5 min), and 80% A (8 min), with a 2 min re-equilibration to the initial conditions before the subsequent injection, totaling 10 min for the analysis. The sample manager compartment was maintained at 20 °C, and the injection volume was 5 µL. Depending on the highest wavelength of the substances under analysis, the PDA data were recorded at 280, 320, and 360 nm. The entire UHPLC system was controlled, and data were gathered using the Empower software 2.0 (Waters, Milford, MA, USA). By contrasting the retention periods and UV spectra with those obtained for pure standards under the same experimental circumstances, the target analytes were identified. Three separate analyses were performed on each extract.

### 2.9. Validation of the µ-QuEChERS/UHPLC-PDA Method

To verify the method’s suitability for the quantification of the target analytes in the investigated samples, the µ-QuEChERS/UHPLC-PDA method was validated in terms of selectivity, linearity, limit of detection (LOD), limit of quantification (LOQ), precision (intra-day and inter-day, expressed as percentage of relative standard deviation (%RSD)), and accuracy (expressed as percentage of recovery, %Rec). Selectivity was determined by comparing the sample extracts to the standard solutions using the µ-QuEChERS/UHPLC-PDA method. The lack of interferences in the retention time and wavelength of the target analytes demonstrated the selectivity of the recommended method. The linearity of a method is measured by how closely a calibration plot of response (typically the chromatographic area of the peak) versus concentration estimates a straight line [35]. The linearity of the µ-QuEChERS/UHPLC-PDA method was established by creating a calibration curve with seven concentration levels. The sensitivity of the µ-QuEChERS/UHPLC-PDA method was evaluated through the calculation of LOD and LOQ. The LOD is the lowest concentration of an analyte from which its identification in a sample may be determined. Likewise, the LOQ is the minimum concentration of an analyte that may be measured in a sample within a limit of confidence. The LOD and LOQ of the µ-QuEChERS/UHPLC-PDA method were obtained using the following formulas: (3.3 × Sb)/a and (10 × Sb)/a, respectively, where Sb refers to the standard deviation of the ordinate at the origin and a is the slope of the calibration curve.

Precision can be categorized as repeatability, intermediate precision, and reproducibility (interlaboratory precision), and it should be evaluated on homogeneous samples [36]. In this study, precision was measured in terms of repeatability, which was achieved by completing a series of repeated analyses in a short period (intra-day), and intermediate precision, which was achieved by repeating the analyses on various, non-consecutive days (inter-days). Intra-day precision was evaluated through the analysis of three extractions in duplicate (n = 6) for each spiking level, whereas inter-day precision was calculated by analyzing three different extractions in triplicate (n = 9) for each spiking level. The precision is given as %RSD. Accuracy was measured by evaluating the analyte recovery in the spiked sample [36]. The accuracy was assayed by spiking *Eugenia uniflora* L. orange fruit at the following three different concentration levels: low, medium, and high.

### 2.10. Statistical Analysis

MetaboAnalyst 6.0 was used for statistical analysis, which includes data pre-processing to normalization (data transformation utilizing data scaling by mean-center and cubic root). To identify significant differences between samples, the normalized data were analyzed by employing one-way ANOVA followed by Turkey’s test for post hoc multiple comparisons of means. The combination of these methods ensured the robust identification of significant differences in the biological activities of *Eugenia uniflora* L. leaf and fruit extracts, since one-way ANOVA is an important test for significant differences in means across more than two groups under the assumption that data are approximately normally distributed and variances are homogeneous upon normalization, while Tukey’s test controls the family-wise error rate in determining specific group differences, thus making it suitable for pair-wise comparisons in multi-group datasets.

## 3. Results and Discussion

The key results of our study on the assessment of the biological properties of *Eugenia uniflora* L. leaf and fruit extracts related to the prevention of CVDs are presented in the following sections.

### 3.1. Evaluation of Total Polyphenols, Flavonoids, and Anthocyanins from Extracts of Leaves and Fruits of Eugenia uniflora L.

**Figure 5:**
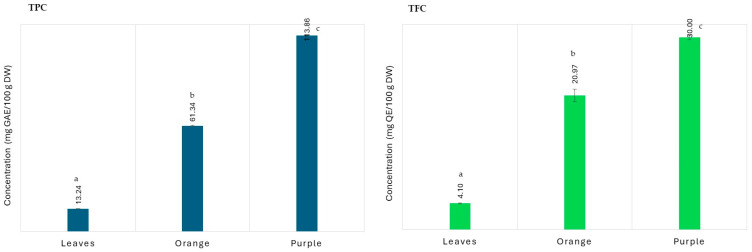
TPC and TFC values determined in *Eugenia uniflora* L. leaf and fruit extracts. Different superscript letters indicate statistically significant differences (*p* < 0.05) among the extracts.

TPC was determined using the Folin–Ciocalteu method. The Folin–Ciocalteu reagent (phosphomolybdenum/phosphotungsten complex), which exhibits a yellow coloration, interacts with phenolic compounds for the determination of TPC by transferring electrons to the complex, resulting in a blue coloration [37]. The TPC results for the *Eugenia uniflora* L. leaf and fruit extracts are presented in Figure 5. The TPC values were 13.2 ± 0.01 mg GAE/100 g DW for the *Eugenia uniflora* L. leaf, 61.3 ± 0.03 mg GAE/100 g DW for the orange pitanga, and 113.9 ± 0.01 mg GAE/100 g DW for the purple pitanga. These values may be explained by the presence of anthocyanins in the purple pitanga.

The TFC assay was carried out using the AlCl_3_ colorimetric method. This method evaluates the approximate number of flavonoids in a sample. In this reaction, a complex is formed between the carbonyl and hydroxyl groups of the flavonoid and the aluminum ion (Al^3+^), giving rise to a yellowish color; the more flavonoids in the matrix, the darker the solution [38]. As can be seen in Figure 5, the TFC value for the *Eugenia uniflora* L. leaf was 4.1 ± 0.01 mg QE/100 g DW, for the orange pitanga, it was 20.9 ± 0.10 mg QE/100 g DW, and the purple pitanga, it was 30.0 ± 0.03 mg QE/100 g DW. Like the TPC, all of the extracts displayed statistically significant differences (*p* < 0.05) among them. In brief, the purple pitanga demonstrated higher values in both the TPC and TFC assays than the orange variety. The values obtained were slightly lower than those reported in the literature, which may be explained by the solvent used in the extraction procedure and by the composition of pitangas, which is influenced by the geographic region of their production, climatic conditions, maturation stage, and variety. In a study carried out by Rodrigues et al. [39], orange pitanga fruit pulp showed a TPC value of 231 mg GAE/100 g using the ultrasound-assisted extraction method. Other comparable results of 179.0 and 201.8 mg GAE/100 g were obtained by Jacques et al. [40] and Bagetti et al. [41], respectively, for orange pitanga pulp. One of the reasons for the discrepancies in the results of these assays could be because the extraction techniques employed in each of these investigations differed from those employed in this investigation.

The TAC was determined by the pH differential method described by Sudarat [33]. When the pH of anthocyanins changes, their pigments undergo reversible structural changes that show up as remarkably distinct absorbance spectra. At pH 1.0, the colorful oxonium form is more common, whereas at pH 4.5, the colorless hemiketal form is more prevalent. Based on this process, the differential pH approach makes it possible to detect the total anthocyanins quickly and accurately, even when polymerized deteriorated pigments and other interfering substances are present. The purple pitanga (155 ± 0.26 mg C3GE/100 g DW) had an anthocyanin content eight times higher than that found in the orange pitanga (18.6 ± 0.18 mg C3GE/100 g DW). In the *Eugenia uniflora* L. leaves, no anthocyanins content was determined. Also, in the TAC assay, the values were below those reported. Oliveira et al. [42] also determined the TAC in red pitanga, obtaining a value quite similar (172 ± 0.05 mg/100 g) to that seen in this study for purple pitanga. Differences in variety and *terroir* may explain these differences.

### 3.2. Evaluation of the Biological Activities from Extracts of Leaves and Fruits of Eugenia uniflora L.

#### 3.2.1. Antioxidant Activity

**Figure 6:**
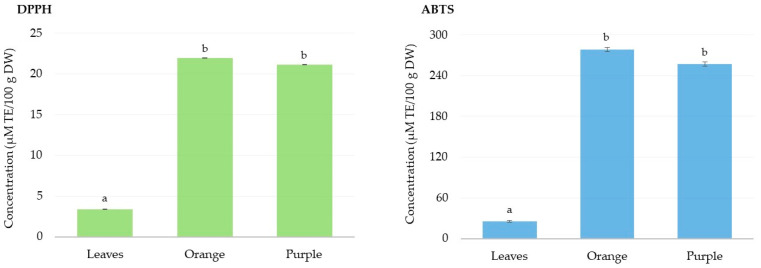
Antioxidant activity of *Eugenia uniflora* L. leaves and fruit (orange and purple) extracts determined by DPPH and ABTS assays. Different superscript letters indicate statistically significant differences (*p* < 0.05) among the extracts.

The antioxidant activity of the investigated *Eugenia uniflora* L. samples (leaves, and orange and purple fruits) were estimated using the DPPH and ABTS assays. These two assays utilize synthetic radicals that are not clearly correlated with food and biological systems, and for this reason, objections are often raised regarding their use. Nonetheless, their affordability, reasonable reproducibility, and ease of use make them widely used. In addition, the literature shows that DPPH and ABTS are the synthetic radical tests most used. The DPPH test is an overly sensitive technique for determining how well sample antioxidant defenses can scavenge the DPPH free radical. This approach is based on the donation of hydrogen atoms from the antioxidant to the radical and the transfer of electrons from the radical to the antioxidant. As it is decreased, its distinctive hue vanishes, making the solution lighter [43]. The obtained values for the DPPH assay, shown in Figure 6, were 3.40 ± 0.01 μM TE/100 g DW for the *Eugenia uniflora* L. leaves, 21.9 ± 0.01 μM TE/100 g DW for the orange pitanga, and 21.1 ± 0.01 μM TE/100 g DW for the purple pitanga. The statistical analysis showed that the DPPH scavenging activity of the two varieties of fruits did not present statistically significant differences.

The ABTS assay was also performed to measure the antioxidant activity of the *Eugenia uniflora* L. leaves and fruits. The ABTS solution has a green coloration. Polyphenols reduce their free radicals, and the greater the reduction in these radicals, the clearer the solution becomes, thus classifying the antioxidant activity of the samples under analysis [44]. Figure 6 shows the obtained results for the *Eugenia uniflora* L. leaves (25.5 ± 1.42 μM TE/100 g DW), orange pitanga (278.5 ± 3.41 μM TE/100 g DW), and purple pitanga (257.4 ± 3.23 μM TE/100 g DW).

The two varieties of fruits showed no statistically significant differences (*p* > 0.05) in terms of the antioxidant activity evaluated by the ABTS assay, however, the ABTS values obtained for the leaves were statistically different from those obtained for the orange and purple pitanga. All analyzed samples showed values for the DPPH assay lower than those obtained from the ABTS assay. According to research conducted by Ferreira et al. [45], *Eugenia uniflora* L. leaf extracts showed values of 78.5 µM TE/g extract for the DPPH assay and 76.7 µM TE/g extract for the ABTS assay. These values are slightly higher than those obtained in the current study, which may be explained by the solvent used in the extraction procedure and the extraction procedure itself. Moreover, the study carried out by Figueira et al. [32] reported that pitanga seeds present a higher antioxidant activity, based on the DPPH and ABTS assays, than lemon (*Citrus limon* var. *eureka*), tangerine (*Citrus reticulata* var. *setubalense*), tomato (*Solanum lycopersicum* var. *gordal*), and uva-da-serra (*Vaccinium padifolium* Sm.).

#### 3.2.2. Antihypertensive Activity

**Figure 7:**
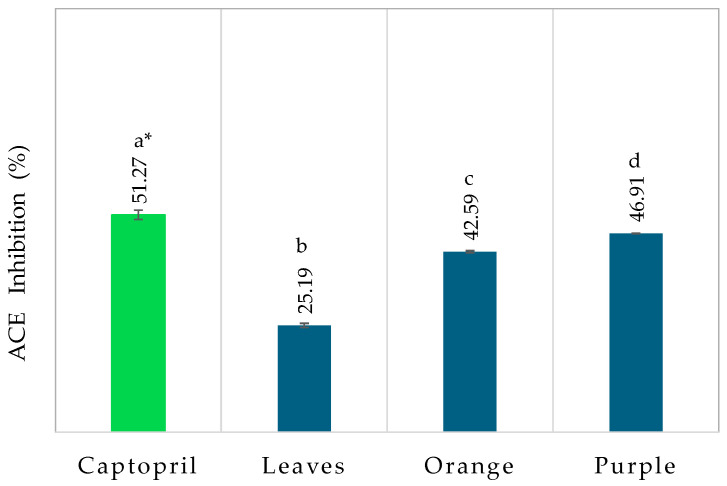
Antihypertensive activity of *Eugenia uniflora* L. leaf and fruit extracts. Different superscript letters denote statistically significant differences (*p* < 0.05) among the extracts. *: reported on work of Nakagawa et al. [48].

Antihypertensive activity was evaluated using the ACE activity inhibition assay (Figure 7). ACE is an enzyme that converts angiotensin I to angiotensin II, which presents as an effective vasoconstrictor, indicating that it narrows blood vessels and increases blood pressure. The purple and orange fruits had an acceptable inhibition rate. The purple variety had a slightly higher inhibition rate than the orange variety, with 46.9 ± 0.11% and 42.6 ± 0.18%, respectively. In the case of *Eugenia uniflora* L. leaves, the inhibition rate decreased by half to around 25.2 ± 0.48%, indicating a decreased ability to block the ACE enzyme. In a study performed by Figueira et al. [32], orange pitanga showed around 90% enzyme inhibition, a value slightly higher than that obtained in this study. The study carried out by Sensu et al. [46] on red barberries showed an inhibition of 73.8%. Das et al. [47] conducted a study evaluating the ACE inhibition activity in various fruits. More than 75% ACE inhibition was shown by aqueous fruit extracts from the red form of *Trapa bispinosa*, *Phoenix sylvestris*, *Cicca acida*, *Achras sapota*, and *Averrhoa carambola*. Conversely, nearly 50% inhibition was shown by *Ziziphus mauritiana*, *Spondias pinnata*, *Trapa bispinosa* (green), and *Punica granatum*. Low activity (<50% inhibition) was demonstrated by *Aegle marmelos*, *Annona squamosa*, *Annona reticulata*, *Feronia elephantum*, *Physalis peruviana*, and *Syzygium jambos* [47]. Nevertheless, according to Nakagawa et al. [48], captopril showed an ACE inhibition activity of 63%, generally used as a control, demonstrating to be more efficient compared to *Eugenia uniflora* L. leaf and fruit extracts.

#### 3.2.3. Anti-Inflammatory Activity

**Figure 8:**
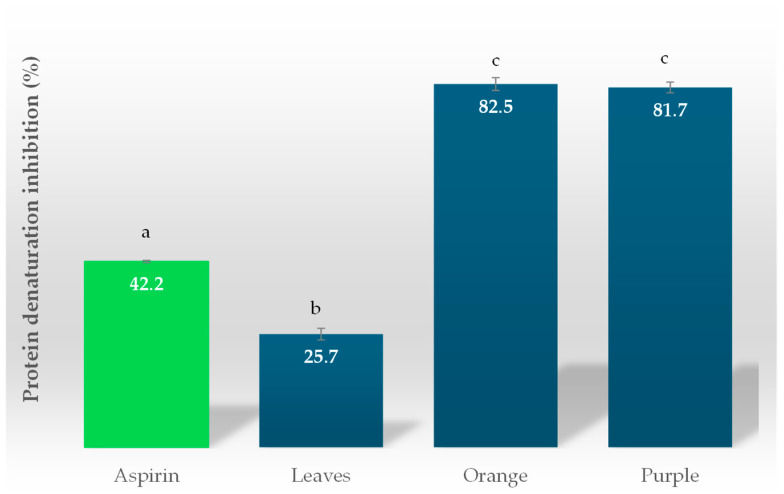
Anti-inflammatory activity of *Eugenia uniflora* L. leaves and orange and purple fruit extracts. Different superscript letters denote significant differences (*p* < 0.05) among the extracts.

The protein denaturation inhibition assay was used to measure the anti-inflammatory activity (Figure 8) in the leaves and purple and orange fruits of *Eugenia uniflora* L. Protein denaturation occurs when a protein loses its native structure and becomes unfolded, which can lead to a loss of function. This assay is useful for identifying molecules that can stabilize proteins and prevent denaturation, which is important in various biological processes. This would suggest that the higher the percentage of inhibition of protein denaturation, the greater the anti-inflammatory activity of the extracts. *Eugenia uniflora* L. leaves (25.7 ± 1.29%) had a lower inhibition percentage than the orange pitanga, which had the highest protein denaturation inhibition percentage (82.5 ± 1.53%), similar to the purple pitanga (81.7 ± 1.21%). Aspirin was used as a control, which had a higher percentage of inhibition than the leaf extracts but was lower than the fruits of *Eugenia uniflora* L, as shown in Figure 5. However, the literature indicates that anti-inflammatory agents should suppress protein denaturation by at least 20% [49]. In this way, both varieties of pitanga have a good ability to inhibit protein denaturation, which leads to a greater anti-inflammatory power with the activity to prevent diseases. Gomathi et al. [50] carried out an anti-inflammatory assay to evaluate raspberry extracts for the prevention of diseases driven by inflammatory processes. The obtained results showed that the raspberry extracts had a protein denaturation inhibition of 77%. The study of Lehfa et al. [51] evaluated the anti-inflammatory activity of *Arbutus unedo* L., an evergreen plant belonging to the *Ericaceae* family, observing a percentage of protein denaturation inhibition of 70.1 ± 0.7%.

### 3.3. Validation of the µ-QuEChERS/UHPLC-PDA Method for Polyphenols Analysis

To determine the suitability of the µ-QuEChERS/UHPLC-PDA method for measuring the polyphenols in the *Eugenia uniflora* L. leaves and fruits, the figures of merit of the method, selectivity, linearity, and LOD, LOQ, precision, and accuracy, were determined. Table 1 shows the figures of merit of the optimized methodology.

Selectivity was evaluated by comparing the chromatograms and PDA spectra obtained for the target *Eugenia uniflora* L. extracts to those of analytical standards, without interferences in the RT at which the target analyte was eluted, demonstrating that the approach was selective and allowed for their identification in the *Eugenia uniflora* L. extracts. Linearity was verified for all the compounds within the concentration range studied. The coefficients of determination (R^2^) were greater than 0.994, indicating an acceptable fit of the attained value to the calibration curve. The LOD and LOQ values represent the lowest concentrations at which analytes can be identified and quantified in a sample, respectively. The obtained LODs ranged from 0.011 mg/L (*trans*-resveratrol) to 0.139 mg/L (gallic acid), while the LOQ values ranged between 0.033 mg/L (resveratrol) and 0.421 mg/L (gallic acid), as shown in Table 1. Comparable results can be viewed in previous works [52], suggesting that the proposed methodology is suitable for the quantification of trace levels of these polyphenols in *Eugenia uniflora* L. extracts.

The precision and accuracy of the µ-QuEChERS/UHPLC-PDA method were also verified by spiking orange pitanga at three different concentration levels (Table 2). 

Precision was used to verify the method’s ability to generate reproducible results. It was evaluated in terms of repeatability (intra-day) and intermediate precision (inter-day), with the obtained data expressed as %RSD. The results obtained for repeatability and intermediate precision were less than 20%, showing that the methodological approach performed well on these parameters. The accuracy results are represented as a percentage of recovery (Rec%). The procedure becomes increasingly accurate as the value approaches 100%. Analytical methods typically have an acceptable limit of ±25% accuracy [53]. The accuracy values for the analytes employed in this study (Table 2) varied from 75% (syringaldehyde) to 117% (quercetin). The validation results indicate that the µ-QuEChERS/UHPLC-PDA methodology is a useful analytical approach to extract and analyze the target polyphenols from *Eugenia uniflora* L. leaves and fruits.

### 3.4. Analysis of Polyphenols from Eugenia uniflora L. Leaves and Fruits by µ-QuEChERS/UHPLC-PDA Method

The *Eugenia uniflora* L. samples (leaves and orange and purple fruits) were extracted, identified, and quantified using the µ-QuEChERS/UHPLC-PDA method, which has been demonstrated to be effective for this purpose. Table 3 shows the concentration of each polyphenol identified in the samples. The chromatographic areas for the polyphenols were obtained using the maximum wavelength of each compound, as follows: 280 nm for gallic acid and cinnamic acid, 320 nm for syringaldehyde and resveratrol, and 360 nm for quercetin and kaempferol. The polyphenol profiles changed depending on the variety of pitanga. In orange pitanga, it was possible to identify gallic acid (13.1 ± 0.5 mg/100 g DW), *trans*-resveratrol (1.72 ± 0.01 mg/100 g DW), quercetin (2.23 ± 0.03 mg/100 g DW), and cinnamic acid (0.26 ± 0.19 mg/100 g DW).

Gallic acid (7.19 ± 0.51 mg/100 g DW) and quercetin (0.27 ± 0.02 mg/100 g DW) were also identified in the purple pitanga, but two other polyphenols, syringaldehyde (0.09 ± 0.01 mg/100 g DW) and kaempferol (0.63 ± 0.41 mg/100 g DW), were identified in this variety. In the case of the *Eugenia uniflora* L. leaves, only gallic acid (2.78 ± 0.04 mg/100 g DW) was identified. In a study carried out by Schumacher et al. [24], gallic acid was quantified in *Eugenia uniflora* L. by HPLC, obtaining different concentrations according to the used solvent as follows: 640 ± 2.2 mg/100 g for aqueous extract, 736 ± 1.2 mg/100 g for ethanol extract, and 267 ± 3.2 mg/100 g for methanol/acetone extract. Migueis et al. [29] identified 12 compounds, such as cyanidin-3-hexoside, myricetin-hexoside, and quercetin rhammoside, through HPLC-DAD-ESI/MS. The different results found can be explained by the fact that different solvents and different analysis methods were applied.

#### Properties of Polyphenols Identified in the Leaves and Fruits of *Eugenia uniflora* L.

The results show that the orange and purple pitanga and *Eugenia uniflora* L. leaves contain gallic acid, which is a valuable compound for human health and industrially relevant due to its antioxidant potential. Gallic acid is widely used as a UV radiation protector, an astringent in cosmetics, and a food preservative [54]. Concerning the influence of this compound on the cardiovascular system, some works have shown that gallic acid can have protective effects against cardiotoxicity and arrhythmias [55].

In orange pitanga, it was possible to identify *trans*-resveratrol, quercetin, and cinnamic acid. *Trans*-resveratrol has a wide range of health benefits, which explains the extensive *in vivo* and *in vitro* studies carried out. It presents antioxidant and anti-inflammatory properties, helping in protection against oxidative stress and inflammation [56], antiglycation properties, by inhibiting the formation of advanced glycation end products, which are correlated with aging and diabetic problems [57], antimicrobial properties against a diverse range of bacteria, viruses, and fungi [58], and, in addition, anticancer properties [59]. Moreover, resveratrol has been investigated for its potential in the prevention of various CVDs, including atherosclerosis, hypertension, stroke, myocardial infarction, and heart failure [60].

Quercetin is a flavonoid that occurs naturally in fruits, vegetables, and juices. It has several remarkable properties, such as antioxidant and anti-inflammatory properties, acting as a free radical scavenger and inhibiting lipid peroxidation [61], in addition to anticancer and neuroprotective properties [62]. Quercetin has also been associated with cardiovascular health through decreases in blood pressure and cholesterol levels, and also with improving endothelial function [63].

Another polyphenol identified in orange pitanga was cinnamic acid, which also has some biological properties, such as anti-inflammatory, antimicrobial, anticancer, and antidiabetic properties [64,65]. Cinnamic acid also has hepatoprotective and neuroprotective properties [64]. Cinnamic acid has been shown to have cardioprotective effects, particularly in diabetic cardiomyopathy, and can help to improve heart function, reduce inflammation, and alleviate dyslipidemia [65].

Syringaldehyde and kaempferol were identified in purple pitanga. Syringaldehyde is a natural aromatic aldehyde that has antifungal, antibacterial, and anticancer properties, making it a promising compound for several therapeutic applications. It has also been shown to exert neuroprotective effects, reducing cell damage, increasing antioxidant activity, and inhibiting apoptosis. Kaempferol has been associated in different reports with antioxidant and anti-inflammatory properties, helping to protect against oxidative stress and inflammation [66]. This compound has been shown to have anticancer activity against various types of cancer, as well as neuroprotective effects [67]. It acts also on cardiovascular health, as it is a potential free radical scavenger and inhibits lipid peroxidation, helping to reduce the risk of CVDs [68].

## 4. Conclusions

The TPC, TFC, and TAC values of the investigated leaf and fruit extracts, determined by UV–Vis, showed that the maximum levels were obtained for the purple pitanga (113.7 mg (GAE)/100 g DW, 30.0 mg (QE)/100 g DW, and 154.73 mg (TA)/100 g DW, respectively) and the minimum levels were found for the leaves (13.2 mg (GAE)/100 g DW, 4.10 mg (QE)/100 g DW, and no anthocyanins found, respectively). The extracts of *Eugenia uniflora* L. leaves and fruits had a higher scavenging activity for the ABTS radical compared to DPPH, with the highest value recorded for ABTS (278.5 μM(TE)/ 100 g DW) for the orange pitanga and the lowest for leaves (25.5 μM(TE)/100 g DW). In the ACE inhibition assay, the purple pitanga showed an ability to inhibit this enzyme by 46.9%, which is a good indicator of its promising antihypertensive activity, followed closely by the orange pitanga (42.6%) and leaves (25.2%). In the protein denaturation inhibition assay, the fruit extracts showed optimum inhibition of around 82%, displaying a potent anti-inflammatory activity, significantly outperforming the leaf extracts (26%).

The validation results indicate that the µ-QuEChERS/UHPLC-PDA method effectively extracted and quantified the polyphenols in the *Eugenia uniflora* L. samples (leaves and fruits). The method is selective, since there were no interferences in the RT where the target analytes were eluted. Linearity was also verified for all the target analytes within the concentration range studied. The R^2^ results were greater than 0.994, indicating an acceptable fit of the value obtained to the calibration curve. The LODs obtained ranged from 0.011 mg/L (trans-resveratrol) to 0.139 mg/L (gallic acid), while the LOQ values ranged from 0.033 mg/L (trans-resveratrol) to 0.421 mg/L (gallic acid). The results for repeatability and intermediate precision were less than 20%, showing that the approach performed well in these parameters. The accuracy values for the analytes used in this study ranged from 75% (syringaldehyde) to 117% (quercetin), which is within acceptable limits. Six polyphenols (gallic acid, syringaldehyde, trans-resveratrol, quercetin, cinnamic acid, and kaempferol) were identified in the investigated samples using the µ-QuEChERS/UHPLC-PDA method. Across all the samples, gallic acid emerged as the predominant polyphenol across all samples, with orange pitanga containing the highest concentration (13.1 ± 0.51 mg/100 g DW).

These results highlight the potential of *Eugenia uniflora* L., particularly its fruits, as a valuable source of bioactive compounds, with antioxidant, antihypertensive, and anti-inflammatory properties, suggesting potential for the prevention of CVDs and other chronic conditions, which are leading to global health burdens. The findings support its potential use in functional foods, nutraceuticals, and pharmaceuticals, especially to prevent cardiovascular diseases. The promising results underscore the need for further investigation into the phenolic composition of *Eugenia uniflora* L. to deepen our understanding of its health benefits, since the bioactive potential of *Eugenia uniflora* L. suggests its use as a natural source of antioxidants and biofunctional compounds in functional foods, nutraceuticals, and pharmaceuticals. The presence of potent bioactive compounds, therefore, justifies the agricultural value of *Eugenia uniflora* L. and encourages its cultivation as a high-value crop toward sustainable production systems. This study not only underscores the agricultural value of *Eugenia uniflora* L. but also opens new avenues for its cultivation as a high-value crop in sustainable production systems. Further research is warranted to explore the full therapeutic potential of *Eugenia uniflora* L. and its application in drug development and health interventions.

## Figures and Tables

**Table 1:** Figures of merit of the analytical methodology for μ-QuEChERS/UHPLC-PDA linearity and limits of detection and quantification.

RT (min)	Analyte	λ_max_ (nm)	Concentration Range (mg/L)	Calibration Curve		LOD (mg/L)	LOQ (mg/L)
				Equation	R^2^		
1.60	Gallic acid	280	5–400	y = 2014× + 5948	0.995	0.14	0.4
3.86	Syringaldehyde	320	1–40	y = 11,098× − 8122	0.994	0.02	0.07
5.21	*Trans*-resveratrol	320	3–50	y = 25,342× − 9861	0.997	0.01	0.03
5.92	Quercetin	360	2–20	y = 4333.3× + 3181	0.994	0.07	0.2
6.23	Cinnamic acid	280	6–400	y = 36,651× + 2090	0.997	0.01	0.04
7.21	Kaempferol	360	4–50	y = 13,711× + 12969	0.995	0.02	0.07

RT—retention time; LOD—limit of detection; and LOQ—limit of quantification.

**Table 2:** Precision and accuracy of the μ-QuEChERS/UHPLC-PDA method.

Polyphenols	Spiking Level (mg/L)	Precision (RSD%)		Accuracy (Rec%)
		Intra-Day	Inter-Day	
Gallic acid	5	4.2	19.4	81 ± 4
	100	0.9	4.2	93 ± 6
	400	1.7	3.4	91 ± 0.6
Syringaldehyde	5	0.8	16.6	75 ± 2
	25	2.5	6.9	100 ± 4
	40	3.3	5.7	99 ± 6
*Trans*-resveratrol	3	0.3	14.	11 ± 5
	20	0.5	1.2	94 ± 2
	50	2.3	2.4	95 ± 0.7
Quercetin	4	2.1	8.3	117 ± 8
	10	1.4	3.9	101 ± 9
	20	3.7	3.7	111 ± 2
Cinnamic acid	6	0.4	9.1	93 ± 1
	100	0.3	0.4	91 ± 3
	400	0.4	1.7	96 ± 0.3
Kaempferol	4	0.7	9.6	107 ± 4
	25	0.9	1.9	98 ± 1
	50	3.0	3.4	99± 5

**Table 3:** Results obtained for the identification and quantification of polyphenols in *Eugenia uniflora* L. leaves and fruits through µ-QuEChERS/UHPLC-PDA method.

RT (min)	Analyte	λmax (nm)	Concentration (mg/100 g DW) ± SD		
			Orange Fruit	Purple Fruit	Leaves
1.70	Gallic acid	280	13 ± 0.5	7 ± 0.5	3 ± 0.04
3.86	Syringaldehyde	320	-	0.09 ± 0.01	-
5.21	*Trans*-resveratrol	320	1.7 ± 0.01	-	-
5.90	Quercetin	360	2.2 ± 0.03	0.3 ± 0.02	-
6.23	Cinnamic acid	280	0.3 ± 0.2	-	-
7.20	Kaempferol	360	-	0.6 ± 0.4	-

RT—retention time and SD—standard deviation.

## Data Availability

All data generated or analyzed during this study are included in this article.
